# Photoprotective Potential, Cytotoxicity, and UPLC-QTOF/MS Analysis on Bioactive Solvent Fractions of *Moringa concanensis* Nimmo Bark

**DOI:** 10.1155/2022/3781189

**Published:** 2022-04-23

**Authors:** Rameshkumar Santhanam, Thiruventhan Karunakaran, Kandhasamy Sowndhararajan, Muhammad Faiz Zulkifli, Mouriya Govindan Kothandaraman, Veerasamy Aravindhan, Wan Iryani Wan Ismail

**Affiliations:** ^1^Faculty of Science and Marine Environment, Universiti Malaysia Terengganu, Kuala Nerus 21030, Terengganu, Malaysia; ^2^Biological Security and Sustainability Research Group (BIOSES), Faculty of Science and Marine Environment, Universiti Malaysia Terengganu, Kuala Nerus 21030, Terengganu, Malaysia; ^3^Centre for Drug Research, Universiti Sains Malaysia, Gelugor, Pulau Pinang 11800, Malaysia; ^4^School of Chemical Sciences, Universiti Sains Malaysia, Gelugor, Pulau Pinang 11800, Malaysia; ^5^Department of Botany, Kongunadu Arts and Science College (Autonomous), Coimbatore 641029, Tamil Nadu, India

## Abstract

*Moringa concanensis* Nimmo (Moringaceae) belongs to the same family of *M. oleifera* (miracle tree) and is a medicinal plant traditionally used by Indians to treat various ailments related to diabetes, tumours, inflammation, and blood pressure. Despite its versatility, the photoprotective properties of the plant remain unclear. This study revealed the UV-protective properties of its methanol bark extract and respective subfractions, chloroform, hexane, and ethyl acetate through total phenolic and flavonoid content (TPC & TFC), antioxidant (DPPH), sun protecting factor (SPF) value, and UV absorption spectra analysis. This study also investigated on the inhibitory effect of the tested samples on collagenases and elastase, which are well-known for their role in the skin. The cytotoxic and H_2_O_2_ scavenging properties of *M. concanensis* in 3T3-L1 cells were explored. Finally, the phytochemical profiling of the active fraction was conducted through UPLC-QTOF/MS analysis. Among the tested fractions, the chloroform fraction of *M. concanensis* showed the highest TPC (30.92 ± 0.71 mg GAE/DW), TFC (29.05 ± 0.09 mg QE/DW), and antioxidant properties (IC_50_-6.616 ± 1.90 *μ*gml^−1^). Additionally, chloroform fraction demonstrated the highest SPF value, 10.46 at 200 *μ*gml^−1^, compared to the other tested fractions. All the fractions showed a broad absorption spectrum covering both UVA and UVB ranges. The chloroform fraction of *M. concanensis* also showed collagenase (50%) and elastase (IC_50_-2.95 ± 1.23 *μ*gml^−1^) inhibition properties similar to the positive control. Cytotoxic results revealed that the chloroform fraction of *M. concanensis* prevented the H_2_O_2_-induced oxidative damage in 3T3-L1 cells even at lower concentrations (1.56 *μ*gml^−1^). UPLC-QTOF/MS analysis tentatively identified the presence of bioactive flavonoids and phenolics such as astragalin, quercetin, isoquercetin, and caffeic acid in the active fraction of *M. concanensis* bark. Overall, it is suggested that the chloroform fraction of *M. concanensis* bark has the potency to be used as an active ingredient in sunscreen products.

## 1. Introduction

In recent times, the intensity of ultraviolet (UV) radiation reaching the earth's surface has increased due to ozone depletion by releasing human-made chemicals into the atmosphere. [1]. Three types of UV (200–400 nm) radiations are available: UVA (320–400 nm), UVB (290–320 nm), and UVC (200–290 nm). UV radiation-stimulated skin damage is highly influenced by its wavelength, intensity, and exposure time [2]. UV radiation directly interacts with the skin, which leads to the activation of several signaling cascades in the skin layers. Excessive doses of UVA and UVB rays are widely considered the leading causative agent for sunburns, early aging, DNA damage, wrinkle formation, oxidative stress, and skin cancer. [3]. In particular, UVA radiation produces immediate skin pigmentation and skin alterations related to premature aging *via* penetrating the deeper layers of the skin, whereas UVB is responsible for skin damage, including erythema and sunburn, by inducing late pigmentation. UVB is also associated with changes in cellular DNA and can provoke skin cancer [4]. Both rays are capable of promoting lipid peroxidation, which is strongly associated with the generation of reactive oxygen species (ROS) [5]. The excessive production of ROS leads to direct DNA damage; stimulation of proinflammatory cytokines such as IL-1 (interleukin-1), IL-6 (interleukin-6), and TNF-*α* (tumor necrosis factor); activation of signaling pathways; and the release of matrix metalloproteinases. Overexpression of these biomarkers could damage the matrix proteins such as elastin and collagen, which finally results in phototoxic reactions such as photoallergy, photosensitivity, photoaging, and photocarcinogenesis [6, 7].

Nowadays, sunscreen-based photoprotection is one of the essential strategies utilized to prevent UV-induced damage and other skin-related disorders. Sunscreens can protect the skin from sunburn, sun allergy rash, immune suppression, and other harmful effects induced by UV radiation. These sunscreens are made up of different organic and inorganic filters to protect the skin [3, 4]. Synthetic-based sunscreens are now becoming a serious threat to consumers and a threat to marine lives and the environment. A wide range of novel hypoallergenic cosmetics has been developed using natural products due to the adverse effect of synthetic sunscreen ingredients. Specifically, plant-based products are in high demand due to their myriad benefits such as antioxidant, anti-inflammatory, and immune enhancers [3–9]. Compared to synthetic filters, compounds, fractions, and extracts from plant sources offer a broad range of UV absorption. Several natural products are incorporated into sunscreen products. Previous research reported the usage of numerous traditional plants such as *Moringa. oleifera Lam, Zanthoxylum rhetsa (Roxb.) DC., Parentucellia latifolia Caruel,* and *Nephelium lappaceum L., Camellia sinensis L. Kuntze*, and other plant extracts or fractions that are rich in polyphenols were described to possess appreciable sunscreen properties *via* targeting free radicals, inflammatory pathways (NF-*κ*B, MAPK) and cytokines, and matrix metalloproteinases (MMP1, MMP3, MMP9, etc.) [2, 3, 6, 8, 10, 11]. Though there are studies that revealed the photoprotective potency of some traditional medicinal plants, it remains limited.

*Moringa concanensis* Nimmo is an important traditional medicinal plant in the family of Moringaceae mainly spotted in the Western Ghats of India and also widely distributed in Asian and Arab countries [12]. This species looks almost similar to the well-known medicinal, nutritional value species *M. oleifera*. However, it has some distinct features such as bipinnate leaves, a strong central trunk with an extremely distinctive layer of very furrowed bark, and the petals and sepals of the flower have green patches at the tip. Moreover, the leaves and flowers of *M. concanensis* are larger compared to *M. oleifera* [13]. In Indian traditional systems of medicine, *M. concanensis* is mainly used to treat fertility problems. The leaves and bark of this species were used to treat skin tumours, tiredness, high blood pressure, jaundice, eye problems, diabetes, and swellings [14] with a wide range of therapeutic properties. Studies revealed that the ethanol extract of *M. concanensis* extract exhibited hyperglycaemic activity and showed protective activity against oxidative tissue damage in the pancreas, liver, and kidney [15]. In this study, the photoprotective properties of various solvent fractions of the *M. concanensis* bark were revealed for the first time using biochemical assays such as total phenolic (TPC) and flavonoid content (TFC), DPPH free radical scavenging activity, SPF value determination, and UVA/UVB absorption spectra analysis. Moreover, the inhibitory effect of the plant extract towards collagenase and elastase was also shown using gelatin digestion and antielastase assay. The cytotoxic and H_2_O_2_ damage preventive potential of the extracts and fractions were assessed through the cell culture technique using MTT assay. The potential metabolites present in the active fraction responsible for the biological properties of *M. concanensis* bark were identified through UPLC-QTOF/MS analysis.

## 2. Materials and Methods

### 2.1. Chemical and Reagents

Solvents such as hexane (CAS No. 110-54-3), chloroform (CAS No. 67-66-3), methanol (CAS No. 67-56-1), and ethyl acetate (CAS No. 141-78-6) used for extraction are of analytical grade obtained from Merck Millipore (Darmstadt, Germany). Collagenase from *Clostridium histolyticum* (CAS No. 9001-12-1), N-succinyl-ala-ala-ala-*p*-nitroanilide (AAAPVN, CAS No. 52299-14-6), 2,2-diphenyl-1-picrylhydrazyl (DPPH, CAS, No. 1898-66-4), gelatin (CAS No. 9000-70-8), porcine elastase (CAS No. 39445-21-1), Coomassie blue R-250 (CAS No. 6104-59-2), ascorbic acid (AA) (CAS No. 50-81-7), epigallocatechin gallate (EGCG, CAS No. 989-51-5), Tris-HCl (CAS No. 1185-53-1), agarose (CAS No. 9012-36-6), acetic acid (CAS No. 64-19-7), dimethyl sulfoxide (DMSO, CAS No. 67-68-5), and 3-(4,5-dimethylthiazol-2-yl)-2,5-diphenyltetrazolium bromide (MTT, CAS No. 57360-69-7) were purchased from Sigma-Aldrich Chemicals (St. Louis, MO, USA). Dulbecco's modified Eagle's medium **(**DMEM, CAT No. 11-995-073), TrypLE Express (CAT No. 12604–013), fetal bovine serum (FBS, CAT No. 26-140-079), and penicillin/streptomycin (CAT No. 15140–122) were purchased from Gibco (Life Technologies, California, USA).

### 2.2. Samples Collection and Extraction

The stem bark of *M. concanensis* was collected from Madukkarai, Coimbatore, Tamil Nadu, India. The plant specimen was authenticated, and plant identification certificate was given by Dr. G.V.S. Moorthy, Head of Office, Southern Regional Centre (Letter No. BSI/SRC/5/23/2018/Tech-437), Botanical Survey of India, Coimbatore, Tamil Nadu, India. The Voucher specimen (BUH-VA-3203/2018) was submitted to the Herbarium of Department of Botany, Bharathiar University, Coimbatore, Tamil Nadu, India. The crude methanolic extract (28 g) from dried and powdered bark (600 g) of *M. concanensis* was obtained using ultrasound-assisted extraction technique according to the literature [6].

### 2.3. Liquid-Liquid Partitioning

Various solvent fractions of the crude methanol extract of *M. concanensis* were subjected to liquid-liquid partitioning technique using different solvents based on their polarity. Initially, 15 g of the methanol crude extract was mixed with equal volume of hexane and water (1 : 1) and loaded into the separating funnel. After few agitations, the hexane fraction was removed and the chloroform was added to the separating funnel. Then, the same process was repeated with ethyl acetate. Finally using rotavapor, the resultant fractions such as hexane (3.1 g), chloroform (6.2 g), and ethyl acetate (1.8 g) were obtained and dried under vacuum and lyophilized [11].

### 2.4. Total Phenolic Content

The total phenolic content for all the solvent fractions of *M. concanensis* bark was determined using the Folin–Ciocalteu method [16]. Briefly, 50 *μ*L of the sample (1 mgml^−1^) in methanol was mixed with 50 *μ*L distilled water, 50 *μ*L of 10% Folin–Ciocalteu's phenol reagent, and 50 *μ*L of 1 M sodium carbonate solution in a 96-well microwell plate and incubated for 60 min at room temperature without exposing it to light. Methanol was used as a blank. The absorbance of the reaction mixture was measured using a microplate reader at 750 nm. The total phenolic content was determined through calibration curve using gallic acid (7.81, 15.62, 31.25, 62.5, 125, 250, and 500 *μ*gmL^−1^) as standard. Results are expressed as milligram gallic acid equivalents (GAE) per gram of dry plant extract. All tests were done in triplicates.

### 2.5. Total Flavonoid Content

The total flavonoid content for all the fractions of *M. concanensis bark* was determined using spectrophotometric method [16]. 100 *μ*L of the plant extract (1 mgmL^−1^) and standard solutions of quercetin (7.81, 15.62, 31.25, 62.5, 125, 250, and 500 *μ*gmL^−1^) in methanol solution were mixed with 100 *μ*l of 2% AlCl_3_ solution. Reaction mixtures were incubated for an hour at room temperature. The absorbance was measured using Tecan Infinite F200 Pro plate reader at *λ*max 415 nm. Total flavonoid contents were expressed as mg quercetin equivalent (QE) per gram of dry plant extract. Experiments were done in triplicates.

### 2.6. DPPH Scavenging Activity Assay

The crude extract and the fractions of *M. concanensis* bark were tested for their free radical scavenging properties using DPPH free radical scavenging assay [16, 17]. Briefly, 0.12 mM DPPH in methanol was prepared and mixed with various concentrations of test samples at 1 : 1 ratio (100 *μ*L). Ascorbic acid was used as the positive control. Test samples and DPPH solution were incubated at room temperature for 30 min in the dark. After the incubation, the absorbance value of the mixture was recorded on a microwell plate reader at 570 nm. The experiment was done in triplicates. The DPPH radical scavenging activity was calculated using the formula:

(1) $$\begin{array}{c} \text{DPPH radical scavenging activity}\left(\text{\%}\right)=\frac{\left[\left({\text{Abs}}_{\text{control}}-{\text{Abs}}_{\text{sample}}\right)\right]}{\left[\left({\text{Abs}}_{\text{control}}\right)\right]}\times 100, \end{array}$$

where Abs_control_ is the absorbance of DPPH radical + methanol and Abs_sample_ is the absorbance of DPPH radical + samples/positive control.

### 2.7. SPF Value Determination

The *in vitro* SPF value of the crude extract and solvent fractions of *M. concanensis* were obtained by the method described in the literature [16]. Briefly, the absorbance value of the test sample (200 *μ*gmL^−1^) was determined on a UV-Visible spectrophotometer at 5 nm intervals within the range of 290–320 nm. The SPF value was then calculated by using the formula:

(2) $$\begin{array}{c} \text{SPF spectrophotometric}=\overset{320}{\underset{290}{\text{CF}}}x\sum \text{EE}\left(\lambda \right)xI\left(\lambda \right)x\text{Abs}\left(\lambda \right), \end{array}$$

where CF is the correction factor (= 10), EE (*λ*) is the erythemal effect spectrum, *I* (*λ*) is the solar intensity spectrum, Abs (*λ*) is the absorbance of test sample, where EE (*λ*) *x* I(*λ*) are constants. Methanol was used as a blank, and measurements were made in triplicate.

### 2.8. UVA/UVB Absorption Spectra

The UV absorption spectrum of the crude extract and solvent fractions of *M. concanensis* (100 *μ*gmL^−1^ in methanol) were measured over a wavelength range of 200–400 nm on a UV-Visible spectrophotometer using a quartz cell (1 cm). The UV absorption spectrum of the samples tested was compared with the positive control, epigallocatechin gallate (EGCG) prepared with the same concentration.

### 2.9. Gelatin Digestion Assay

The gelatin digestion assay was done according to the method described by Santhanam et al. [6] with slight modifications. Agarose (2%) was prepared in a collagenase buffer (50 mM Tris-HCl, 10 mM CaCl_2_, 0.15 M NaCl, pH 7.8) and porcine gelatin (0.15%). The mixture was transferred into a petri dish and left at room temperature for 1 h for solidification. Later, the wells were made using a sterile 200 *μ*L microtip. Then, 25 *μ*L of the samples was incubated with 25 *μ*L of bacterial collagenase-1 (0.1 mgmL^−1^) for 1 h. Finally, the reaction mixture (50 *μ*L) was loaded into the well and further incubated overnight. EGCG was used as a positive control. The next day, the petri dish was visualized by the Coomassie brilliant blue staining method. The degree of gelatin digestion was determined by measuring the area of the light translucent zone formed after destaining over a blue background.

### 2.10. Antielastase Assay

The antielastase activity of the various solvent fractions of *M. concanensis* was determined according to the methods of Santhanam et al. [6], with minor modifications. The assay was performed in Tris-HCl buffer (0.2 mM, pH 8.0). The stock solution of porcine pancreatic elastase (PE–E.C. 3.4.21.36) was dissolved in sterile water. N-succinyl-ala-ala-ala-p-nitroanilide (AAAPVN) was dissolved in 1 mL Tris-HCl (0.2 mM) buffer (pH 8.0), and the samples were dissolved in buffer. Then, the test samples and elastase were incubated for 15 minutes before adding the substrate to start the reaction. The final reaction mixture contains a buffer, 0.8 mM AAAPVN, 50 *μ*gmL^−1^ PE, and the test samples at various test concentrations in a total volume of 250 *μ*L. EGCG served as the positive control while water served as the negative control. The absorbance value was measured using a Tecan Infinite F200 Pro plate reader (Tecan Group Ltd., Männedorf, Switzerland) at 410 nm.

(3) $$\begin{array}{c} \text{Enzyme inhibition activity }\left(\%\right)\;=\frac{\left[\left({\text{Abs}}_{\text{control}}-{\text{Abs}}_{\text{sample}}\right)\right]}{\left[{\text{Abs}}_{\text{control}}\right]}\times 100. \end{array}$$

### 2.11. Cell Culture

3T3-L1 cells were maintained in complete Dulbecco's modified Eagle's medium (DMEM) high-glucose media according to the method of Raseetha et al. [18] with slight modification. The media were supplemented with 10% fetal bovine serum (FBS) and 5% of penicillin-streptomycin in a humidified 5% CO_2_ incubator at 37°C. Once the cell reaches 70–80% confluence, it was utilized for seeding and treatment.

#### 2.11.1. Cytotoxicity

Cells cytotoxicity was performed using MTT assay based on the method described by Raseetha et al. [18]. The cells were seeded at a density of 1 × 10^5^ cells/well in 96-well plates. Once the cell reaches 80% confluence, the 100 *μ*L of various concentrations of methanol extract and solvent fractions of *M. concanensis* and positive control EGCG were treated in each well. After 24 h, 20 *μ*L of MTT was added to the cells and it was incubated at 37°C for 4 h. The medium was replaced with 100 *μ*L DMSO, and the absorbance for each well was measured at 570 nm on Tecan Infinite F200 Pro plate reader. Cytotoxicity assay was conducted to determine the range of concentrations of the active fraction. Experiments were performed in triplicates.

#### 2.11.2. Protective Effect of Samples against H_2_O_2_-Induced Oxidative Damage in 3T3-L1 Cells

The protective effect of *M. concanensis* fractions against H_2_O_2_ was performed according to Chen et al. [19] with slight modification. The 3T3-L1 cells were seeded on 96-well plates at a density of 1 × 10^5^ (in 100 *μ*L medium) per well and incubated at 37°C for 24 h. Once the cell reaches the confluence, the medium was replaced with 100 *μ*l of samples with various concentrations (0–100 *μ*g mL-1) and positive control (ascorbic acid) for 18 h. Then, the cells were treated with H_2_O_2_ (125 *μ*M) to induce oxidative damage, and the dose concentration was fixed based on the current study. After 6 h, the medium was removed, and the cells were washed thrice with PBS. Next, the MTT solution was added and the cells were further incubated for 4 h. Finally, the formazan crystals were dissolved in DMSO (100 *μ*L per well), and the absorbance value was measured at 540 nm using Tecan Infinite F200 Pro plate reader [20].

### 2.12. Ultraperformance Liquid Chromatography Quadrupole Time-of-Flight Mass Spectrometry (UPLC-QTOF/MS) Analysis

Phytochemical profiling in chloroform fractions of *M. concanensis* was conducted via ultraperformance liquid chromatography (UPLC-MS) analysis. The analysis was carried out using Waters ACQUITY ultraperformance LC system (Waters, Milford, MA, USA). Chromatographic separation of the respective extract was conducted using a selected column (ACQUITY UPLC HSS T3, 100 mm × 2.1 mm × 1.8 m, Waters, Manchester, UK) maintained at 40°C. The UPLC systems were connected to Vion IMS QTOF detector (Waters, Milford, MA, USA). The mobile phases used in the analysis were 0.1% formic acid (*A*) and acetonitrile (B). The composition of mobile phases was consisted of the following multistep linear gradient: 0 min, 1% B and 99% A; 0.5 min, 1% B and 99% A; 16.00 min, 35% B and 65% A; 18.00 min, 100% B and 0% A; and 20.00 min, 1% B and 99% A, respectively. The injection volume used for the sample was 1 *μ*L while the flow rate was set at 0.6 mL/min. The data were obtained from the UHPLC system coupled with Vion IMS QTOF hybrid mass spectrometer from waters, equipped with a LockSpray ion source. The ion source was operated in negative electrospray ionization (ESI) mode by applying specific conditions such as the capillary voltage at 1.50 kV, reference capillary voltage at 3.00 kV, source temperature at 120°C, desolvation gas temperature at 550°C, desolvation gas flow at 800 L/h, and cone gas flow at 50 L/h, respectively. Nitrogen (>99.5%) was used as desolvation and cone gas. Data were acquired in high-definition MSE (HDMSE) mode ranging between *m/z* 50 and 1500 at 0.1 s/scan. Hence, two independent scans with different collision energies (CE) were alternatively obtained during the run: a low-energy (LE) scan at a fixed CE of 4 eV and a high-energy (HE) scan where the CE was ramped from 10 to 40 eV. Argon (99.999%) was used as collision-induced-dissociation (CID) gas. Data interpretation was carried out using Waters UNIFI Scientific Information System database. Resolved peaks were further identified with the assistance of reported values from previous literature and are shown in Table 1.

### 2.13. Statistical Analysis

All the data were represented as mean ± SD. Statistical analyses were performed using GraphPad Prism version 5 with one-way ANOVA followed by Dunnett's test. *p* values < 0.05 were considered to be statistically significant.

## 3. Results and Discussion

### 3.1. Total Phenolic and Flavonoid Contents

It is well established that phenolic compounds from various plants are strongly associated with their biological properties. Hence, it would be worthwhile to determine the total phenolic and flavonoid contents in the plant extracts [10]. The total phenolic and flavonoid contents of methanol extract and its fractions of *M. concanensis* bark are presented in Table 2. The total phenolic (30.920 ± 0.71 mg GAE/g DW) and flavonoid (29.054 ± 0.09 mg QE/g DW) contents were found to be higher in the chloroform fraction followed by hexane fraction. Phenolic components are regarded as powerful antioxidants and act in redox-sensitive signaling cascades to prevent DNA damage [11].

Data expressed as mean ± SD, *n* = 3. Data with different alphabet superscript letters show significant difference at *p* < 0.05, among different solvents, with multiple comparisons (one-way ANOVA, followed by Dunnett's test).

### 3.2. Antioxidant Activity of *M. concanensis* Bark Fractions

#### 3.2.1. Free Radical Scavenging Assay

**Figure 1:**
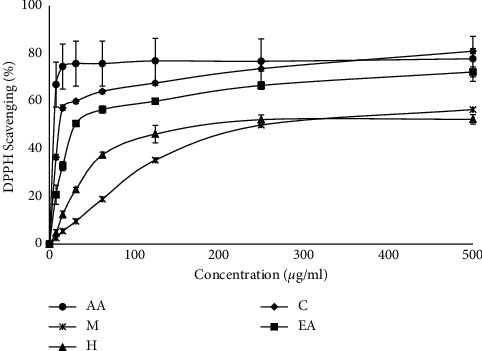
DPPH radical scavenging activity of the methanol extract and its fractions from the bark of *M. concanensis*. AA, ascorbic acid; M, methanol extract; H, hexane extract; C, chloroform extract; EA, ethyl acetate extract. (*n* = 3).

The free radical scavenging capacity of the crude methanol extract and other fractions of *M. concanensis* bark were determined using the DPPH assay, and the results are shown in Figure 1. All the samples exhibited varying degrees of concentration-dependent DPPH radical scavenging activity. In that, the chloroform fraction showed the strongest DPPH radical scavenging activity with the IC_50_ value of 6.616 ± 1.90 *μ*gmL^−1^ followed by ethyl acetate fraction (IC_50_ = 13.4 ± 2.22 *μ*gmL^−1^) which is comparable to the positive control, ascorbic acid (AA). Gori et al. [22] revealed the photoprotective effect of 11 medicinal plants *Atalantia ceylanica (Arn.) Oliv, Argyreia populifolia Choisy*, *Hibiscus furcatus Roxb. ex DC*, *Ipomoea mauritiana Jacq*, *LAsia spinosa (L.) Thw*, *Leucas zeylanica (L.) W.T.Aiton*, *Olax zeylanica Wall., Ophiorrhiza mungos Linn.*, *Plectranthus amboinicus (Lour.)*, and *Mollugo cerviana (L.).Ser.* where the plant that showed high SPF values is closely related to its antioxidant properties. Numerous studies also suggested that the plant fraction which shows high free scavenging properties offers significant protection towards UV-induced damage [16, 22].

### 3.3. Protective Effect of the *M. concanensis* Fractions against UV

#### 3.3.1. Sunscreen Protection Factor (SPF) Value

SPF value refers to the efficacy of test samples that protects the development of UV radiation-induced erythema [23]. It is a measure of UVB protection offered by sunscreen products. As the value increases, the percentage of UVB protection increases. As shown in Table 3, at the concentration of 200 *μ*gmL^−1^, the chloroform fraction of *M. concanensis* bark exhibited the highest SPF value (10.50 ± 0.23 with >87% of UVB protection) compared to the other fractions such as ethyl acetate and hexane. The SPF value of the most active ingredient epigallocatechin gallate (EGCG) from *Camellia sinensis* (Green Tea) is 10.34 ± 0.21, which is almost like the SPF value of the chloroform fraction of *M. concanensis* bark. The literature revealed that the seed oil from *M. concanensis* could be a good candidate for sunscreen formulation [24]. The bark extract of medicinal plants such as *Curatella americana* L and *Amburana cearensis (Allemao) A.C. Sm.* showed SPF values 14.74 and 12.21 at 0.2 mgmL^−1^ which is suggested to be the promising source of future skin care products [25]. Similarly, the bark extract of *M. concanensis* could also be utilized as one of the encouraging ingredients in natural product sunscreen formulation.

#### 3.3.2. UV Absorption Spectra

**Figure 2:**
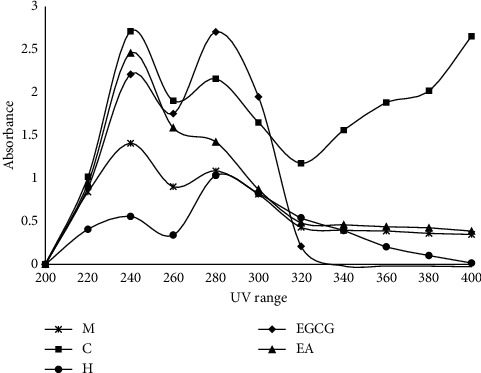
UV absorption spectra of the methanol extract and its fractions from the bark of *M. concanensis*. M, methanol extract; C, chloroform extract; H, hexane extract; EGCG, epigallocatechin gallate; EA, ethyl acetate extract (*n* = 3).

UV absorption spectra of various solvent fractions of *M. concanensis* and the positive control EGCG are shown in Figure 2. The results showed that all the fractions of *M. concanensis* have a wide range of UV absorption; however, among the other fractions, the chloroform fraction of *M. concanensis* showed a broad spectrum of UV absorption which covers UVA (320–400 nm), UVB (280–320 nm), and also UVC (200–280 nm). The positive control EGCG showed the absorption under the UVB range only. Similar patterns of results were observed in the active fraction of *Zanthoxylum rhetsa* (*Roxb*.) *DC.* bark extract which is suggested to be utilized in broad-spectrum sunscreen formulations [6]. Compared to the active fraction of *Z. rhetsa*, the UV absorption spectra of *M. concanensis bark* are high. At present, several studies and regulatory bodies recommend using broad-spectrum natural sunscreen to prevent the damages such as wrinkling and sunburns induced by both UVA and UVB, respectively [26]. The results revealed that the chloroform fraction of *M. concanensis* bark could be an optional ingredient to be used in a natural broad-spectrum sunscreen formulation.

### 3.4. Gelatin Digestion Assay

Collagenases are one of the important matrix metalloproteinases responsible for the breakdown of collagen present in the skin layers. Studies revealed that continuous exposure of UV radiation could increase the level of collagenases that might damage the matrix proteins and cause wrinkle formation as well as lead to premature aging [27]. The collagenase inhibitory activity of extract and various solvent fractions of *M. concanensis* are presented in Table 4. Compared to the extract and other fractions, the chloroform fraction showed appreciable collagenase inhibitory activity of 50% at the concentration of 200 *μ*gmL^−1^ which is comparable to that of EGCG. However, the hexane and ethyl acetate fractions exhibited a weaker collagenase inhibition activity. Studies revealed that various parts of medicinal plants such as leaves extracts (methanol) of *Aegle marmelos* (*L*.) *Correa, Acalypha indica Linn, Calotropis gigantea* (*L.*) *W.T. Aiton, Nerium oleander* L., and *Nyctanthes arbor-tristis Linn* and a rhizome extract of *Acorus calamus. Linn* at the concentration of 1 mgmL^−1^ showed 50% collagenase inhibitory activity [28], whereas the ethyl acetate fraction of the bark extract of *Z. rhetsa* showed 50% collagenase inhibition at the concentration of 500 *μ*gmL^−1^ [6].

### 3.5. Antielastase

**Figure 3:**
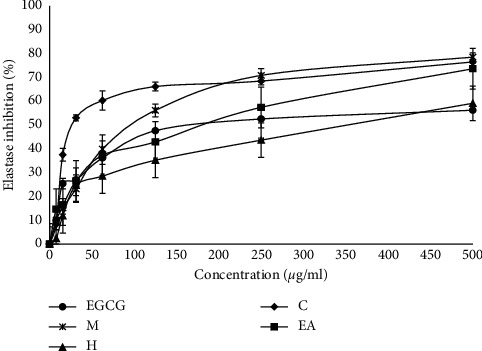
Antielastase activity of the methanol extract and its fractions from the bark of *M. concanensis.* Data are expressed as mean ± SD, *n* = 3.

Elastase is a serine protease, responsible for the breakdown of elastin, an important protein responsible for skin elasticity [29]. The inhibitory activity of methanol extract and its fractions of *M. concanensis* on elastase enzyme were examined.  Figure 3 and Table 5 show the effect of methanol extract and its fractions of *M. concanensis* bark against elastase enzyme activity. Among different fractions tested, the chloroform fraction exhibited remarkable elastase inhibition activity with the IC_50_ value of 2.957 ± 1.23 *μ*gmL^−1^ than the standard EGCG (IC_50_-39.32 ± 1.41 *μ*gmL^−1^). These findings demonstrated that chloroform fraction of *M. concanensis* may have antiaging potentials by inhibiting elastase enzyme production and delaying the degradation of elastin fibers. Researchers suggested that the plant fraction that is rich in phenolics and flavonoids is reported to possess significant antielastase activity. The ethyl acetate fraction of *Garcinia daedalanthera Pierre.* stem bark extract possesses 43.96 ± 12.53% elastase inhibitory effect at the concentration of 100 ppm [30]. In this study, the chloroform fraction of *M. concanensis* bark exhibited >50% of elastase inhibitory effect at the concentration of 31.25 *μ*gmL^−1^.

### 3.6. Cytotoxicity of *M. concanensis* Fractions in Cell Culture (3T3-L1 Cell Lines)

#### 3.6.1. MTT Assay

**Figure 4:**
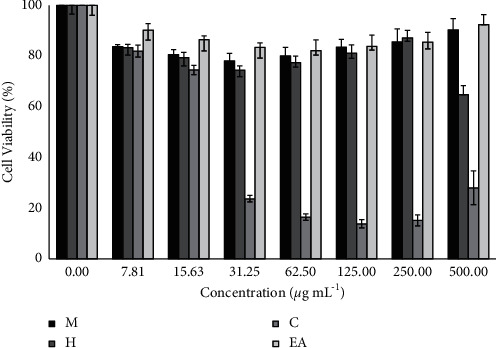
Cytotoxic effect of the methanol extract and various solvent fractions of *M. concanensis* bark treated with 3T3 mouse fibroblasts for 24 h data expressed as mean ± SD, *n* = 3.

Cytotoxic effect of various solvent fractions of *M. concanensis* was tested in 3T3-L1 cells with a wide range of concentration (0–500 *μ*gmL^−1^). Results revealed that all the fractions of *M. concanensis* bark except the chloroform fraction were nontoxic to the cells up to 250 *μ*gmL^−1.^ However, the chloroform fraction of *M. cocanensis* was toxic to the cells at the concentration >25 *μ*gmL^−1^, where it showed the strongest cytotoxic activity against 3T3-L1 cells by reducing the cell viability to 16.5% at the concentration of 62.5 *μ*gmL^−1^, Figure 4. This is the first study to reveal the cytotoxic effect of various solvent fractions of *M. concanensis* bark against the preadipocyte cells. Previously, the ethanol extract of the leaves of *M. concanensis* was also reported to be nontoxic to 3T3-L1 adipocytes up to 100 *μ*gmL^−1^ until 24 h of treatment [15]. Balamurugan et al. [31] tested the crude ethanol extract of *M. concanensis* leaves and bark against HepG2 cell lines and revealed that both parts of *M. concanensis* strongly inhibit the growth of cancerous cells in a dose-dependent manner. In another study, the methanolic root bark extract of *M. concanensis* was found to be toxic against HepG2 cells, whereas it does not induce toxic effect against A549 and HT-29 cell line which demonstrates its selective cytotoxicity [21]. In this study, the crude methanol extract of *M. concanensis* bark does not induce any toxicity against 3T3-L1 cells up to 500 *μ*gmL^−1^. Since the other solvent fractions of *M. concanensis* bark have toxic effect >250 *μ*gmL^−1^ and the chloroform fraction has toxic effect >25 *μ*gmL^−1^, on an average, we reduced the concentration range of all the extracts/fractions to 0–100 *μ*gmL^−1^ for further assay.

#### 3.6.2. H_2_O_2_-Induced Damage

**Figure 5:**
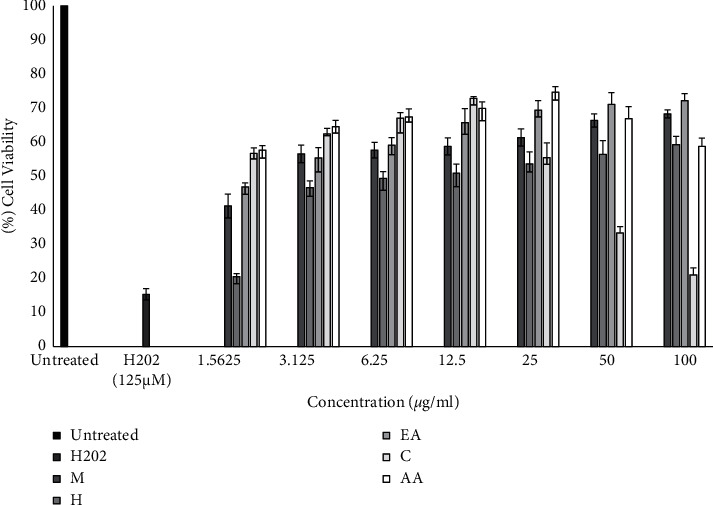
Protective effect of various solvent fractions of *M. concanensis* bark against H_2_O_2_-induced oxidative damage in 3T3-L1 cells treated for 24 h. Data are expressed as mean ± SD, *n* = 3. Data represent significant difference at *p* value < 0.05, among different solvent fractions of *M. concanensis* bark (various concentrations) compared with the H_2_O_2_ (125 *μ*M), using one-way ANOVA, followed by Dunnett's test.

The protective effect of various solvent fractions of *M. concanensis* bark against H_2_O_2_-induced oxidative damage in 3T3-L1 was determined and shown in Figure 5. From the results, it has been clear that the H_2_O_2_ (125 *μ*M) reduced the viability of cells to 15.35% after 6 h of treatment, whereas the cells pretreated with various solvent fractions of *M. concanensis* bark prevented the cells from oxidative damage. However, the level of protection by each fraction varies and it depends on the concentration and constituents present in each sample. The chloroform fraction of *M. concanensis* bark offers significant protection against H_2_O_2_-induced damage even at low concentration, where the viability of cells treated with 1.56 *μ*gmL^−1^ protects 56% of cells which was comparable to that of positive control, AA (cell viability—57%). As the concentration increases, up to 12.5 *μ*gmL^−1^, the chloroform fraction prevented the cells up to 72% where AA prevented up to 69%. However, at the concentration of 25 *μ*gmL^−1^ and above, the viability of cells treated with chloroform fraction got drastically reduced. This might be due to the overdose of chemical constituents present in it. Similar patterns of results were obtained in the cytotoxicity assay, and at low concentration (<15 *μ*gmL^−1^), the chloroform fraction was nontoxic to 3T3-L1 cells. As the concentration increases, the chloroform fraction is toxic. As reported, the extracts and fractions of various parts of *M. concanensis* are cytotoxic to different cancer cell lines like HepG2 [31, 32], and the chloroform fraction of *M. concanensis* bark could offer a significant cytotoxic effect in other cancerous cell lines. Further research is needed to evaluate the selective cytotoxic effect of chloroform fraction of *M. concanensis* bark.

From this study, it has been evident that compared to other fractions, the chloroform fraction of *M. concanensis* bark has a high sunscreen protection factor value, broad-spectrum UV absorption, appreciable DPPH scavenging, anticollagenase, antielastase, high phenolic, and high flavonoid content. Additionally, it also prevents the H_2_O_2_-induced oxidative damage in 3T3-L1 cells which was comparable to that of positive control, AA.

### 3.7. UPLC-QTOF/MS Analysis

From this study, it has been identified that the chloroform fraction is the bioactive fraction of *M. concanensis* bark. UPLC-QTOF/MS analysis was found to be a convenient method to determine the presence of possible phytoconstituents in the studied fraction through the exact mass detection. Polyphenolic compounds such as phenolic acids and flavonoids are naturally abundant and commonly found in the genus *Moringa*. Bioactive flavonols such as quercetin, hyperoside, astragalin, isoquercetin, kaempferol-3-*O*-rutinoside, and rutin were reported previously in this genus especially in the species, *Moringa oleifera* [20]. In this study, 20 different phytoconstituents were identified in the chloroform fraction obtained from the methanol extract of *M. concanensis* bark using UPLC-QTOF/MS analysis, Table 1. The result indicated that the chloroform fraction contains a complex mixture of bioactive tentative annotated phenolics such as kaempferol-3-*O*-rutinoside, astragalin, quercetin-3-*O*-glucuronide, kaempferol 3-*O*-glucuronopyranosyl methyl ester, isoquercetin, kaempferol-3-*O*-arabinoside, kaempferol-3-*O*-rhamnoside, quercetin-3-*O*-glucuronide-methyl ester, eugenol, quercetin, and caffeic acid. The assignations were carried out on a prediction basis and detailed phytochemistry studies required to be carried out on this species for the affirmation of the presence of the predicted metabolites in the studied fraction/extract in the future. This comprehensive phytochemical profiling analysis has revealed the structural diversity of phenolics and their similar structural patterns as well as the biological potency of the studied fractions.

Moreover, the findings are in agreement with the results obtained from the total phenolic and total flavonoids evaluation conducted on the respective fractions. The existence of flavonoids, especially flavonols, could possibly be the main contributors to photoprotective properties especially in collagenase and elastase inhibitory activities through a synergistic effect [33]. Furthermore, the presence of carbonyl and hydroxyl moieties in the main scaffold of the flavonols might be played the role in giving the inhibitory effects which allow them to bind to metalloenzymes like collagenase and elastase that could alter or inhibit metabolic pathways [34]. However, further studies are required in relation to the detailed identification and isolation of bioactive metabolites from the bark of *M. concanensis* as well as molecular mechanisms of their photoprotective potentials.

## 4. Conclusion

In summary, the result obtained from the phytochemical analysis showed that chloroform fractions of *M. concanensis* have the highest DPPH, TPC, and TFC values. The photoprotective study also demonstrated a high SPF value for chloroform fractions and gave the same UVB protection as its positive control, EGCG. Besides that, the chloroform fractions have a much broader spectrum of UV absorption that covers both UVA and UVB when compared to others. The inhibitory effect of *M. concanensis* chloroform fractions towards elastase and collagenase proved that the plant extract has potential biological properties that can reduce the breakdown of collagen and elastin within the skin. *In vitro* study showed that some of the fractions possessed low cytotoxicity towards the 3T3-L1 cell line even at higher concentrations (methanol, hexane, and ethyl acetate). However, chloroform fractions obtained from the plant extract exhibit high cytotoxic activity even at lower concentrations (ranging from 31.25 to 500 *μ*gmL^−1^). All the fractions displayed protective effect against H_2_O_2_-induced oxidative damage in 3T3-L1 cells, with methanol, hexane, and ethyl acetate having a higher cell viability percentage even at higher concentrations (50 and 100 *μ*gmL^−1^). Various metabolites especially bioactive flavonols that were tentatively identified using UPLC-QTOF/MS analysis might be the source for the high photoprotective activity. Based on these results, *M. concanensis* has the potential to be a new applicant as an active component in pharmaceutical and cosmetics formulation. However, detailed preclinical research is needed to demonstrate the individual or synergistic effect of the bioactive components involved in the photoprotective mechanism of *M. concanensis.*

## Figures and Tables

**Table 1:** Compounds tentatively identified in the chloroform fraction from the methanol extract of *M. concanensis* bark using UPLC-QTOF/MS analysis.

Peak no.	Tentative identification	Elemental composition	Calculated m/z [M-H]^−^	Observed m/z [M-H]^−^	Retention time	References

1	Kaempferol-3-*O*-rutinoside	C_27_H_30_O_15_	593.1507	593.1512	6.65	[21]
2	Astragalin	C_21_H_20_O_11_	447.0927	447.0937	7.50	[21]
3	Quercetin-3-*O*-glucuronide	C_21_H_18_O_13_	477.0670	477.0685	7.83	—
4	Tetra-*O*-galloyl-glucoside	C_34_H_28_O_22_	787.0994	787.1005	8.11	—
5	Kaempferol-3-*O*-glucuronosyl methyl ester	C_22_H_20_O_12_	475.0876	475.0867	8.61	[21]
6	Isoquercetin	C_21_H_20_O_12_	463.0877	463.0884	8.63	[21]
7	Caffeic acid	C_9_H_8_O_4_	179.0344	179.0352	9.01	[21]
8	Hydroxy-methoxy-cinnamic acid	C_10_H_10_O_4_	193.0501	193.0511	9.38	—
9	Sinapic acid	C_11_H_12_O_5_	223.0607	223.0613	10.04	—
10	Kaempferol 3-*O*-arabinoside	C_20_H_18_O_10_	417.0822	417.0844	10.40	—
11	Secoisolariciresinol	C_20_H_26_O_6_	361.1651	361.1656	10.43	—
12	Kaempferol-3-*O*-rhamnoside	C_21_H_20_O_10_	431.0978	431.0995	10.91	[21]
13	Quercetin-3-*O*-glucuronide-methyl-ester	C_22_H_20_O_13_	491.0826	491.0842	10.99	—
14	Quercetin	C_15_H_10_O_7_	301.0348	301.0351	11.93	[21]
15	Eugenol	C_10_H_12_O_2_	163.0759	163.0765	12.47	[21]
16	Naringenin	C_15_H_12_O_5_	271.0607	271.0615	13.07	—
17	Trimethoxyflavone	C_18_H_16_O_5_	311.0920	311.0929	13.32	—
18	Demethoxycurcumin	C_20_H_18_O_5_	337.1076	337.1085	15.53	—
19	Sanleng acid	C_18_H_34_O_5_	329.2328	329.2339	15.62	[21]
20	Licoricone	C_22_H_22_O_6_	381.1338	381.1335	15.65	—

**Table 2:** Total phenolic and flavonoid contents of the methanol extract and its fractions from the bark of *M. concanensis*.

Fractions	Total phenolics mg GAE/g DW	Total flavonoids mg QE/g DW

Methanol	9.858 ± 0.036^d^	3.655 ± 0.061^d^
Hexane	14.306 ± 0.073^b^	9.089 ± 0.193^b^
Chloroform	30.920 ± 0.717^a^	29.054 ± 0.099^a^
Ethyl acetate	12.154 ± 0.158^c^	4.055 ± 0.056^c^

**Table 3:** The sun protection factor (SPF) value and UVB protection effect of the methanol extract and its fractions from the bark of *M. concanensis*.

Samples (200 *μ*gml^−1^)	SPF value	UVB protection (%)

Methanol	4.37 ± 0.17^*∗∗∗*^	>75
Hexane	5.49 ± 0.01^*∗∗∗*^	>75
Chloroform	10.50 ± 0.23^ns^	>87
Ethyl acetate	5.18 ± 0.01^*∗∗∗*^	>75
Epigallocatechin gallate	10.34 ± 0.21	>87

Data are expressed as mean ± SD, *n* = 3. Data represent a significant difference at (^*∗∗∗*^) *p* value < 0.05, among different solvent fractions compared with the positive control (EGCG), using one-way ANOVA, followed by Dunnett's test.

**Table 4:** Gelatin digestion activity of the methanol extract and its fractions from the bark of *M. concanensis.*

Samples (200 *μ*gmL^−1^)	Zone inhibition (mm)	Enzyme inhibition (%)

Control	20 ± 0.81	0
EGCG	10 ± 0.47^*∗∗∗*^	50
Chloroform	10 ± 1.24^*∗∗∗*^	50
Hexane	16 ± 0.47^*∗∗∗*^	20
Ethyl acetate	16 ± 0.47^*∗∗∗*^	20
Methanol	18 ± 0.81	10

Data are expressed as mean ± SD, *n* = 3. Data represent significant differences at (^*∗∗∗*^) *p* value < 0.05, among different solvent fractions compared with negative control (water), using one-way ANOVA, followed by Dunnett's test.

**Table 5:** IC_50_ values of elastase inhibition activity of the methanol extract and its fractions from the bark of *M. concanensis.*

Samples	IC_50_ (*μ*gmL^−1^)

EGCG	39.32 ± 1.41
Methanol	88.53 ± 1.95^*∗∗*^
Hexane	78.11 ± 1.72^*∗*^
Chloroform	2.957 ± 1.23^*∗∗*^
Ethyl acetate	176.7 ± 2.20^*∗∗∗*^

Data expressed as mean ± SD, *n* = 3. Data represent significant differences at *p* value < 0.05, among different solvent fractions compared with the positive control (EGCG), using one-way ANOVA, followed by Dunnett's test.

## Data Availability

Data used to support the findings of this study are obtained from the corresponding author.
